# Comment on “Underreporting of inferior vena cava filter characteristics in diagnostic radiology reports: a call for standardization”

**DOI:** 10.1186/s42155-025-00624-0

**Published:** 2025-11-27

**Authors:** George Rahmani

**Affiliations:** Pacific Radiology Group, 123 Victoria Street, Christchurch, New Zealand

To the Editor,

I commend Wu et al. for their thoughtful letter, which emphasises the importance of accurately documenting inferior vena cava filter (IVCF) characteristics in diagnostic radiology reports [1]. Despite our best intentions and efforts, IVCF retrieval rates remain suboptimal, and every opportunity should be taken to remove these filters where possible, as soon as possible. As the authors highlight, incidental or unrelated imaging provides a valuable opportunity to re-engage these patients and facilitate retrieval.

I would like to clarify a minor point in Fig. 1A, schematic diagram of inferior vena cava models, where the ALN filter is listed among permanent devices. The ALN filter is, in fact, a retrievable system. Both configurations, with and without the hook, are designed to be safely removed using the manufacturer’s proprietary retrieval kits or, for the hook design, with any standard snare-based system available to the operator [2, 3].

This clarification reinforces the authors’ key message: precise identification and reporting of filter type and retrievability are essential to prevent misclassification, missed retrieval opportunities, and long-term device-related complications.

## Data Availability

Not applicable.
